# A case of superficial spreading type of poorly differentiated adenocarcinoma of the stomach with invasion to the esophagus

**DOI:** 10.1186/s12957-022-02605-2

**Published:** 2022-04-29

**Authors:** Junichi Mase, Takahito Adachi, Shunya Kiriyama, Takeshi Horaguchi, Kazunori Yawata, Aiko Ikawa, Bun Sano, Susumu Imai, Kiyohisa Okamoto, Takashi Shiroko

**Affiliations:** 1 Department of Surgery, Japanese Red Cross Takayama Hospital, 3-11 Tenman-machi, Takayama-shi, Gifu 506-8550 Japan; 2 Department of Gastroenterology, Japanese Red Cross Takayama Hospital, 3-11 Tenman-machi, Takayama-shi, Gifu 506-8550 Japan; 3 Department of Pathology, Japanese Red Cross Takayama Hospital, 3-11 Tenman-machi, Takayama-shi, Gifu 506-8550 Japan

**Keywords:** Superficial spreading type of carcinoma of the stomach, Poorly differentiated adenocarcinoma, Adenocarcinoma of the esophagogastric junction

## Abstract

**Background:**

Invasion is more likely to occur in gastric cancer affecting larger areas. Poorly differentiated adenocarcinoma tends to invade deep. The cardiac region prefers submucosal invasion because the submucosa is coarser than the other regions.

**Case presentation:**

A 75-year-old man presented with a chief complaint of abdominal discomfort and weight loss. Esophagogastroduodenoscopy revealed an irregular ulcerative lesion with partial redness of the upper body and lesser curve of the stomach. A continuous shallow depressed lesion invaded the abdominal esophagus by approximately 40 mm. Poorly differentiated adenocarcinomas (por, sig) were observed on biopsy. Grossly, the cancer appeared to extend into the muscle layer; however, we could not confirm invasion into the muscle layer in our biopsy tissue. We diagnosed the lesion as a superficial spreading type of advanced gastric cancer and performed a total gastrectomy, D2-lymph node dissection (spleen preservation), Roux-en-Y reconstruction, and cholecystectomy. Postoperative histopathological examination revealed extensive infiltration of poorly differentiated adenocarcinoma (90 mm × 55 mm), and all were intramucosal lesions. The final pathological diagnosis was T1a, N0, M0, and Stage IA. The postoperative course was uneventful and the patient was discharged on postoperative day (POD) 11. Five years have passed since the operation, and the patient is alive without recurrence.

**Conclusion:**

We encountered a case of gastric carcinoma in which poorly differentiated adenocarcinomas expanded extensively. All lesions were intramucosal.

## Background

Generally, the likelihood of invasion in gastric cancer increases with the area affected [1, 2]. Poorly differentiated adenocarcinoma tends to invade deep [3]. The cardiac region prefers submucosal invasion owing to the submucosa being coarser than that of the other regions [4]. Here, we report a case of an extensively superficial spreading type of poorly differentiated adenocarcinoma of the stomach.

## Case report

**Fig. 1:**
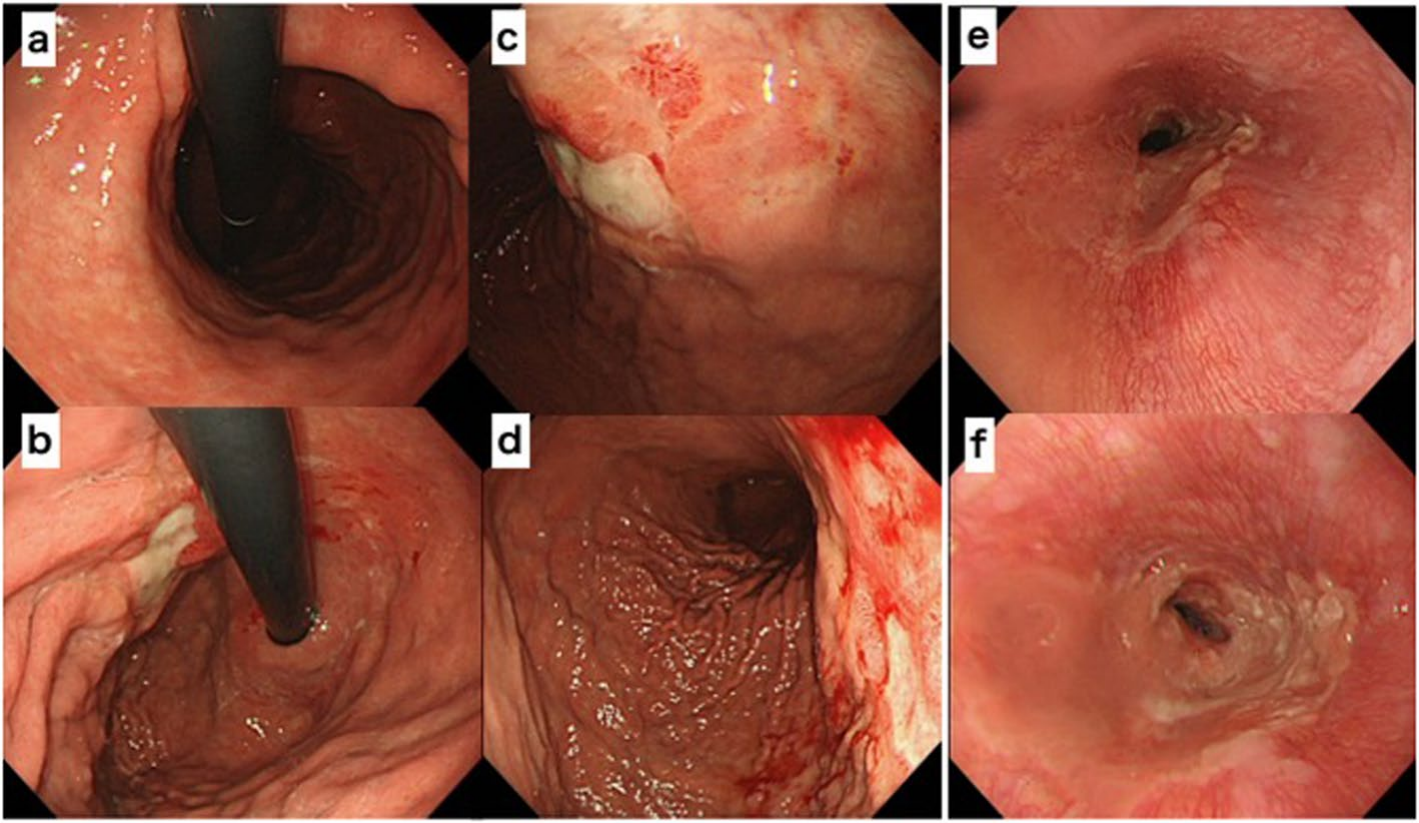
Esophagogastroduodenoscopy. **a**–**d** The widespread shallow depressed lesion extending from the cardia to the mid-body. **e**, **f** About 40 mm esophageal invasion from the esophagogastric junction

**Fig. 2:**
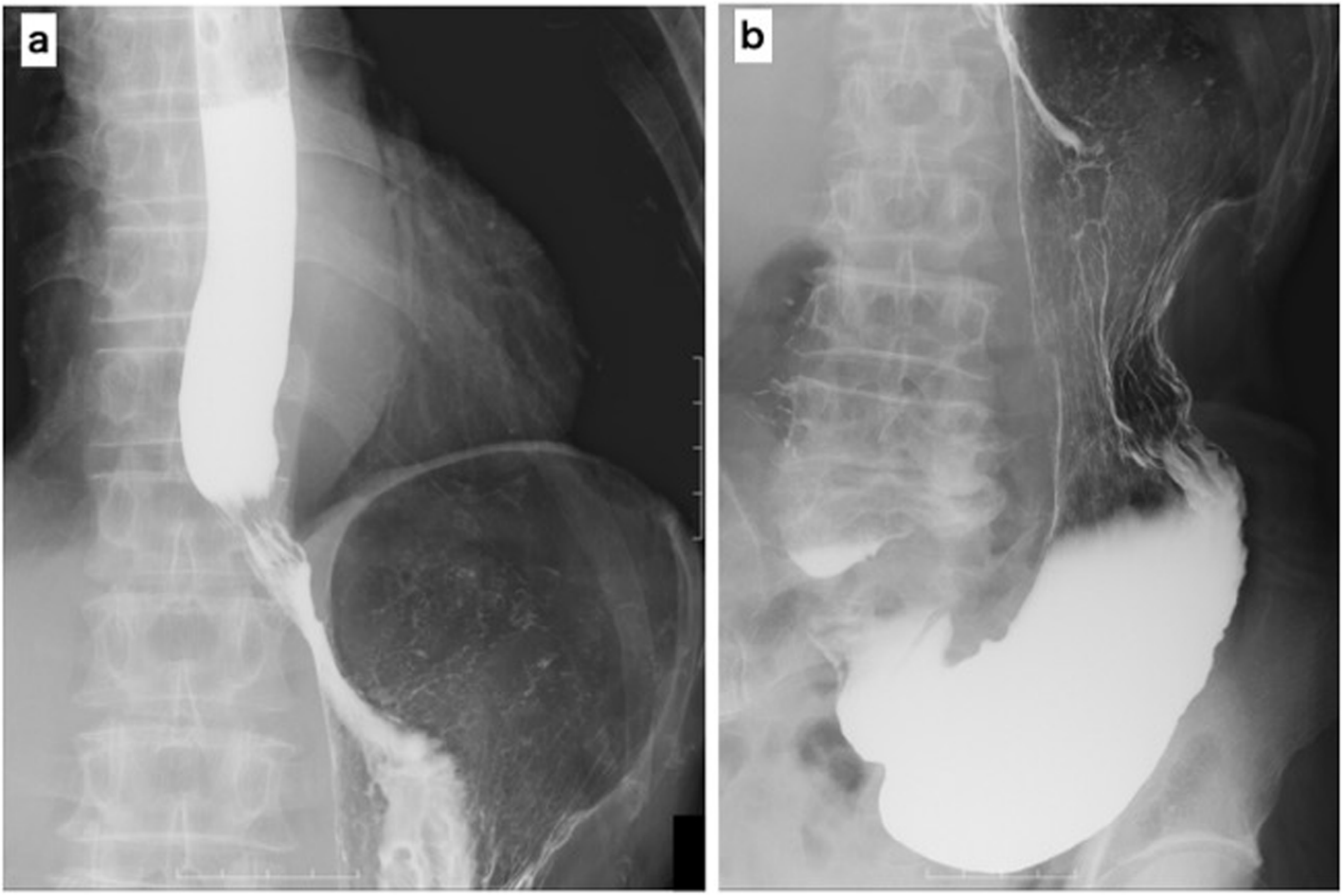
Upper gastrointestinal contrast. **a** There is a storage of contrast agent 40 mm from the esophagogastric junction. **b** A depressed lesion is found just below the cardia

**Fig. 3:**
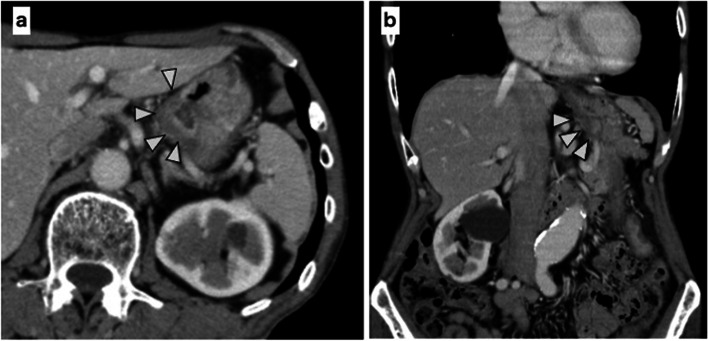
Contrast CT. **a**, **b** Contrast irregularity on the side of the lesser curve of the stomach and increased density of fatty tissue

**Fig. 4:**
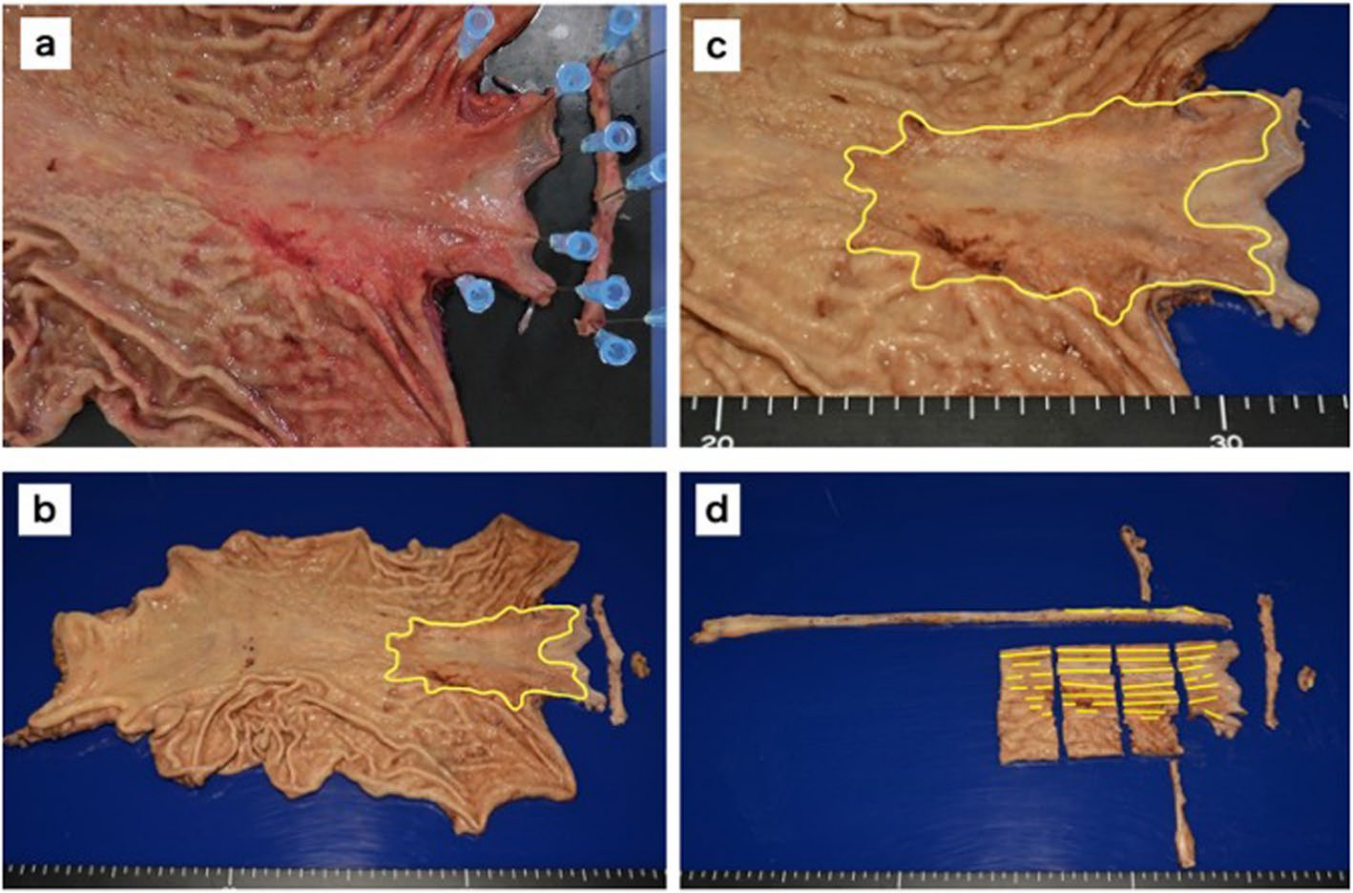
Specimen. **a** After half fixing. **b**, **c** After fixing. **d** After specimen separation. As preoperatively diagnosed, the lesion was a shallow and depressed measuring 90×55 mm from the abdominal esophagus to the upper body and with a lesser curve. The lesion was accompanied by an ulcer

**Fig. 5:**
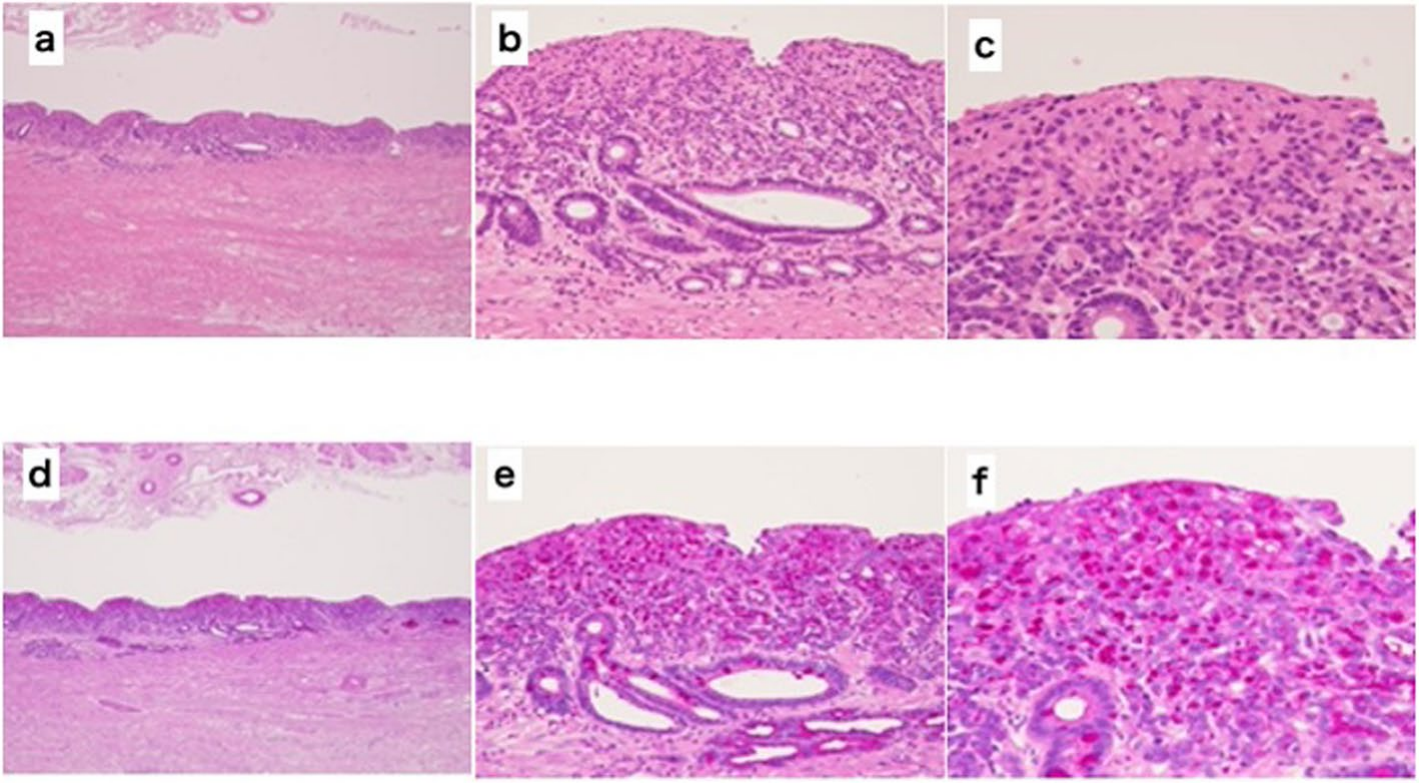
Microscopic image of a specimen. **a** HE stain×40, **b** HE stain×200, **c** HE stain×400, **d** PAS stain×40, **e** PAS stain×200, **f** PAS stain×400. No invasion of the proper muscle layer is observed in any lesion

A 75-year-old man presented with a chief complaint of abdominal discomfort and weight loss. His medical history included hypertension, paroxysmal atrial fibrillation, and refractory pruritus. He had lost approximately 10 kg of weight in 2 months and was referred to our hospital because of abdominal discomfort. At the time of consultation, his height was 174.8 cm, body weight was 56.5 kg, body mass index was 18.49; his vital signs were as follows: body temperature 36.1 °C, blood pressure 128/79 mmHg, heart rate at 77/min, and oxygen saturation at 97 % (room air). Hematological investigations performed on admission revealed carcinoembryonic antigen at 5.1 ng/mL, carbohydrate antigen19-9 at 0.1 U/mL, and carbohydrate antigen − 125 at 7.7 U/mL, indicating no increase in tumor markers. No abnormalities were observed in the blood tests. Esophagogastroduodenoscopy showed a wide, shallow, depressed lesion from the cardia to the mid-body, with an ulcerated part, which we diagnosed as a type 3 tumor (Fig. 1a–d). In addition, approximately 40 mm of the entire circumference of the esophagus was invaded (Fig. 1e, f). The lesions were diagnosed in Group5 (por, sig). Based on the biopsy results, all lesions in the ulcer presented as a wide shallow depressed lesion in the mid-body and lesions at the esophagogastric junction. Upper gastrointestinal examination showed that, on the oral side, lesions had an irregular wall and poor progression up to approximately 40 mm from the esophagogastric junction. On the anal side, they extended around the mid-body (Fig. 2a). We found an ulcerative lesion near the cardia (Fig. 2b) and suspected invasion at the depth of the muscularis propria (MP). Abdominal computed tomography (CT) showed an irregularly thickened wall on the upper body and lesser curvature of the body, and an increase in the concentration of fatty tissue around the wall was also found in the mid-body (Fig. 3a, b). Therefore, the degree of invasion was determined to be at the depth of MP. There were no findings of suspected lymph node enlargement or metastases to other organs. Considering these facts, we preoperatively diagnosed gastric cancer as T2, N0, M0, StageIB (according to the UICC, 8th edition). We performed a total gastrectomy with abdominal esophageal resection, D2 lymph node dissection, cholecystectomy, and Roux-en-Y reconstruction. The proximal side of the invasive esophageal area (40 mm from the esophagogastric junction) was clipped as a marker preoperatively. Intraoperatively, it was confirmed that there was no cancer at the esophageal margin and that it was resected, including the clip. The excised specimen showed a shallow depressed lesion of 90×55 mm from the abdominal esophagus to the lesser curvature of the upper stomach, and a type 0-IIb + IIa + IIc lesion (according to the macroscopic classification of the gastric cancer) [5] with esophageal invasion up to 30 mm from the esophagogastric junction (Fig. 4a–d). Histopathological findings showed that all widespread lesions were cancers that remained in the mucosa, with por2 being the majority and some sig being mixed. No submucosal invasion was observed, including in the ulcerated area (Fig. 5a–f). The pathological diagnosis was gastric cancer: T1a, N0, M0, and StageIA. The postoperative course was uneventful and the patient was discharged on POD 11. Five years since the operation, the patient is surviving without recurrence.

## Discussion

In 1942, Stout named gastric cancer with less deep invasion compared to its size as “superficial spreading type of carcinoma of the stomach [6].” However, he did not clearly define the superficial spreading type of carcinoma of the stomach. Yasui et al. defined it as early gastric cancer of ≧25 cm^2^ [7], and the same definition was adopted in various documents thereafter. This case also had a diameter of 90×55 mm, and the depth of invasion was up to mucosa, thus satisfying the definition of a superficial spreading type of carcinoma of the stomach. Early gastric cancer accounts for 2.5–5.0% of all gastric cancers [8–10].

The superficial spreading type of carcinoma of the stomach has a relatively good prognosis despite its wide range compared with gastric cancers of similar size [6, 7]. On the other hand, it has been reported that among the early cancers, (1) there are a significant number of undifferentiated cancers [10], (2) the rate of submucosal invasion is significantly high [10], and (3) the lymph node metastasis rate is significantly high [9]. Yasui et al. also showed that superficial spreading type of carcinoma of the stomach has a low 5-year survival rate compared to small size gastric cancer [10]. Thus, the superficial spreading type of carcinoma of the stomach is classified as early gastric cancer because of its shallow invasion. However, considering the histological type and lymph node metastasis rate, it is incorrect to define it as early gastric cancer based only on the depth of invasion. In addition, we found literature discussing lymph node metastasis in superficially extensive early-stage gastric cancer. Kyong et al. [11] classified patients into two groups: those with lesions larger than 6 cm and those with lesions smaller than 6 cm, and found that, on multivariate analysis, those with lesions larger than 6 cm were more strongly associated with lymph node metastasis than those with lesions smaller than 6 cm. They concluded that submucosal and lymphovascular invasion are strongly associated with lymph node metastasis, although they were not associated with lymph node metastasis. In the present case, all foci were intramucosal carcinomas, there was no lymphovascular invasion, and no metastasis was found in the dissected lymph nodes. According to the literature, this case falls into the low-risk group for lymph node metastasis. Considering this, we believe that the scope of lymph node dissection can be reduced in patients with superficially extensive early-stage gastric cancer with a low risk of lymph node metastasis.

Notably, in this case, the tissue type was poorly differentiated, in addition to the superficial spreading type of the carcinoma of the stomach. Microscopic photographs of the lesions in the specimen showed that por and sig were present only in the mucosa, and no tub was found. The esophagus also had a por and a sig. Based on histopathological findings, the lesion had thickened walls, which were considered to be due to ulcerations and scar tissue; the mucosa was regenerated at the same site. It was apparent that it took time for the ulcers and scars to form because the mucous membrane had regenerated. It is presumed that it is easier to invade the same layer than penetrate various layers. Therefore, owing to the balance between cancer invasion and tissue regeneration, the superficial spreading type of carcinoma of the stomach takes time to invade. However, it seems easier for cancer cells to invade the same layer.

In addition, it was judged to be a type 3 tumor in this case owing to the large size and level of disorder. Whether the invasion depth can be accurately diagnosed preoperatively remains controversial. The narrow band imaging findings were unstructured, the surface structure was unclear, there was no vascular network, and it was judged that it was a poorly differentiated adenocarcinoma. Endoscopic ultrasound (EUS) was not performed in this case, but it can be used to improve the accuracy of diagnosing the depth of invasion [12]. Endoscopic findings suggest poorly differentiated adenocarcinoma with ulcers and widespread lesions. Therefore, surgery is the 1st choice of treatment, even in cancer cases. EUS was not performed in this case because we suspected deep invasion into the submucosal layer and thought that surgery was necessary. However, when judging the depth of invasion of early gastric cancer using EUS, the misdiagnosis rate was 15.2% and 32.7% according to Ozawa et al. and Kaneko et al., respectively [13, 14]. In other words, even if early gastric cancer is diagnosed using EUS, the surgical method, especially the extent of lymph node dissection for the superficially extended type, should be carefully considered. Considering this report, we should have performed EUS in this case as well, as EUS could accurately estimate the depth of the disease before surgery.

There are two theories of pathogenesis regarding the mechanism of development: monocentric, in which a single lesion spreads extensively, and polycentric, in which multiple lesions coalesce to form a broad lesion. No conclusion has yet been reached [15]. In this case, the pathological findings showed that the gastric mucosa was atrophied to the cardia, so the degree of atrophy can be judged as the open type (O -I) according to Kimura and Takemoto classification [16]. Although biopsy results were negative for H. pylori, atrophic gastritis was widely observed. The lesion was included the extent of atrophic gastritis. Since the mucosa is formed in the ulcer, it is inferred that gastric cancer develops over time through repeated cancer invasion and tissue regeneration. However, it is impossible to decide whether this case is based on monocentrism or polycentrism. The developmental mechanism of superficial spreading type of adenocarcinoma of the stomach requires a further accumulation of cases and studies.

## Conclusion

Gastric cancer is widespread and often invasive. However, it is important to accurately diagnose the depth of invasion using multiple examinations, since the superficial spreading type of adenocarcinoma of the stomach also exists, although it is relatively rare. On the other hand, it requires careful diagnosis and treatment, considering that it is an early gastric cancer but has a worse prognosis than the usual early cancer.

## Data Availability

Not applicable.

## Abbreviations

POD: Postoperative day
MP: Muscularis propria
CT: Computed tomography
EUS: Endoscopic ultrasound
HE: Hematoxylin and Eosin stain
PAS: Periodic acid-Schiff stain
